# Modelling and Analysis of a New Piezoelectric Dynamic Balance Regulator

**DOI:** 10.3390/s121114671

**Published:** 2012-11-02

**Authors:** Zhe Du, Xue-Song Mei, Mu-Xun Xu

**Affiliations:** 1 School of Mechanical Engineering, Xi'an Jiaotong University, Xi'an 710049, China; E-Mails: duzhe.329@stu.xjtu.edu.cn (Z.D.); xsmei@mail.xjtu.edu.cn (X.-S.M.); 2 State Key Laboratory for Manufacturing Systems Engineering, Xi'an Jiaotong University, Xi'an 710049, China

**Keywords:** dynamic model, balance regulator, in-plane bending vibration, motorised spindle, piezoelectric stator

## Abstract

In this paper, a new piezoelectric dynamic balance regulator, which can be used in motorised spindle systems, is presented. The dynamic balancing adjustment mechanism is driven by an in-plane bending vibration from an annular piezoelectric stator excited by a high-frequency sinusoidal input voltage. This device has different construction, characteristics and operating principles than a conventional balance regulator. In this work, a dynamic model of the regulator is first developed using a detailed analytical method. Thereafter, MATLAB is employed to numerically simulate the relations between the dominant parameters and the characteristics of the regulator based on thedynamic model. Finally, experimental measurements are used to certify the validity of the dynamic model. Consequently, the mathematical model presented and analysed in this paper can be used as a tool for optimising the design of a piezoelectric dynamic balance regulator during steady state operation.

## 1. Introduction

As a novel type of piezoelectric actuator, ultrasonic motors have been studied by researchers and companies all over the World for nearly 40 years. These devices use the converse piezoelectric effect of piezoceramics and convert the ultrasonic vibration of the stator into linear or rotational motion in a rotor using the friction force. They inherently posses slow speed/high torque output, high holding torque and rapid response characteristics, all of which combine for the potential to be used as a precise and accurate positioning actuator. Because standing wave-type ultrasonic motors have the advantage of accurate positioning without feedback, a new idea is proposed in this paper, an ultrasonic piezoelectric dynamic balance regulator based on the basic principles of a standing wave-type ultrasonic motor. This regulator is the key component of an *in-situ* dynamic balancing system for a motorised spindle to achieve high precision balancing.

There are many different types of dynamic balance regulators that have evolved over the years in the field of rotor balancing. Van de Vegte [1], who first studied *in situ* dynamic balance regulators for motors in 1964, proposed that a balance block, whose radial position relative to the centre of rotation was under continuous adjustment by a worm gear mechanism, could be used to achieve *in situ* rotor balancing. In 1978, Van de Vegte modified the dynamic balance regulator to use a mass block driven by a motor to change the angular displacement relative to the attached rotating spindle. The electrical power for the driving motor was supplied by external brush contacts [2,3]. Bishop [4] presented a similar design in 1982. Lee *et al.* [5] used a wireless remote-control technique to control a dynamic balance regulator in 1987. However, there were other problems with the balancing motor in addition to the difficulty in power transmission. For example, due to the significant size and inertia of the balancing motor, the dynamic balance system, which rotated with the spindle, could limit the maximum rotating speed and cause imbalance. To solve these problems, in 1998, Zeng *et al.* [6,7] presented a new electromagnetic dynamic balance regulator with a small axial dimension and no moving parts in the dynamic balance system. The approach of rotating a balance mass relative to the spindle was similar to the principle of a stepping motor. Up to this day, two basic types of dynamic balance devices have been used/applied in industrial fields. One type is similar to the electromagnetic dynamic balance devices described; such devices are produced by Dittel in Germany and Lord in the United States. The other typeis the sprayed-fluids dynamic balance device used primarily in grinding machines. This type of device was developed by a German company, Hofmann, in the 1960s and is still produced by some companies.

In this paper, a new type of piezoelectric dynamic balance regulator is introduced, which can overcome some of the disadvantages of existing dynamic balance regulators. As pointed out before, this regulator based on the basic principles of a standing wave-type ultrasonic motor, and combines features such as high driving torque at low rotational speed, high holding torque without an applied electric power, extremely low noise in operation, accurate positioning without feedback, simple mechanical design and rapid response. Because the regulator utilises the in-plane bending vibration mode of the piezoelectric stators to drive the rotor, a contact interface exists on the circumferential side face. This type of structure further minimises the regulator thickness and miniaturises the regulator.Meanwhile, because the regulator is driven with single-phase AC voltage, this type of drive mode simplifies the supply power and enhances the system reliability. Above all, a particularly attractive feature of the regulator is that it possesses high displacement resolution and can achieve control precision on the order of microns, thus it can achieve very high adjustment accuracy.

The topic of this paper is to perform high-efficiency dynamic modelling and to optimise the key parameters of this regulator. Analytical, numerical and experimental methods have been employed in these investigations. For simplicity, the non-linear contact mechanics are studied under the assumption that the vibrational characteristics of the resonating structure are not affected by the contact process. This analysis process simplifies the investigations considerably and quite often results in a good description of the mechanism of motion for the regulator. For an experimental illustration of the theoretical and numerical results, a sample device was fabricated and characterised based on this mechanical analysis. The experimental results reported on here confirm the validity and applicability of the regulator structure and can be used for guidance in further optimisation of the regulator.

In Section 2 of this paper, the overall structural characteristics and the operating principles of the piezoelectric dynamic balance regulator are described in detail. In Section 3, a dynamic analysis on the stator, the rotor, the friction layer and the stator-rotor contact model is performed, which permits the energy conversion characteristics of the regulator to be obtained. In Section 4, MATLAB is employed to numerically simulate the characteristics of the regulator based on the above mathematical model, and optimisation of core parameters for the regulator is conducted according to the simulation results. Afterwards, a prototype is fabricated using the optimised parameters, and its performance is tested experimentally. Comparing the experimental and simulated load torque/rotational speed relation for the regulator, the validity of the dynamic model is proven. Finally, conclusions and future research prospects are detailed in Section 5.

## 2. Overall Structure and Actuation Principle of the Piezoelectric Dynamic Balance Regulator

**Figure 1.**
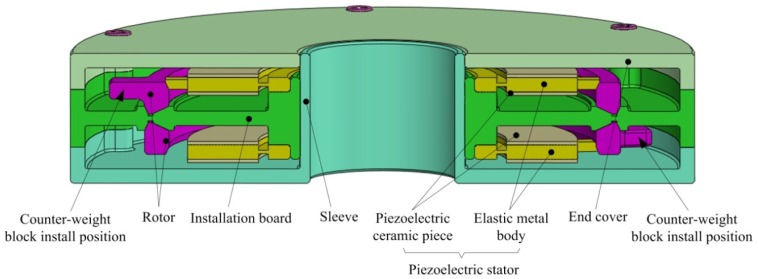
Cross-sectional view of the *in situ* piezoelectric dynamic balance regulator.

Figure 1 shows the structure of the piezoelectric dynamic balance regulator, which can be mounted on the end of the rotor of a motorised spindle system using an interference fit or a keyway to rotate with the rotor. Except for the counterweight block, all of the components of the regulator are axially symmetric to obtain an intrinsically balanced design.

**Figure 2.**
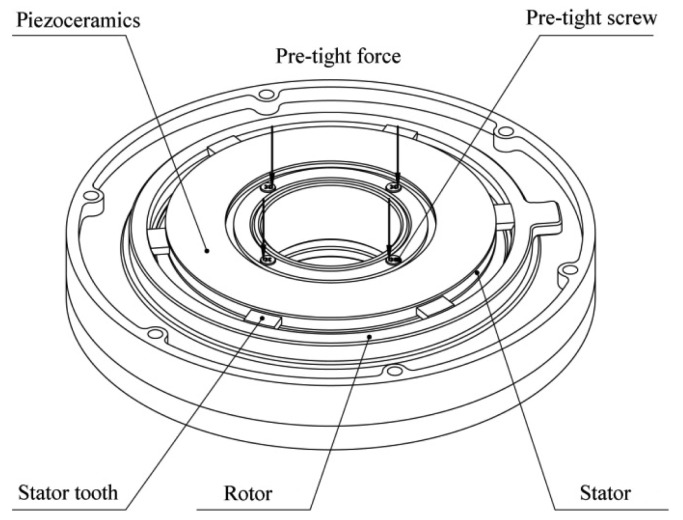
Contact friction drive scheme of the stator and the rotor.

There are two piezoelectric actuating devices installed in the regulator housing. Each device consists of a rotor and a piezoelectric stator with six driving teeth. The stator is fixed on the mounting surface of the regulator. The driving teeth on the stator press against the inner circumference of the rotor, which rotates in a groove on the mounting surface (Figure 2).

**Figure 3.**
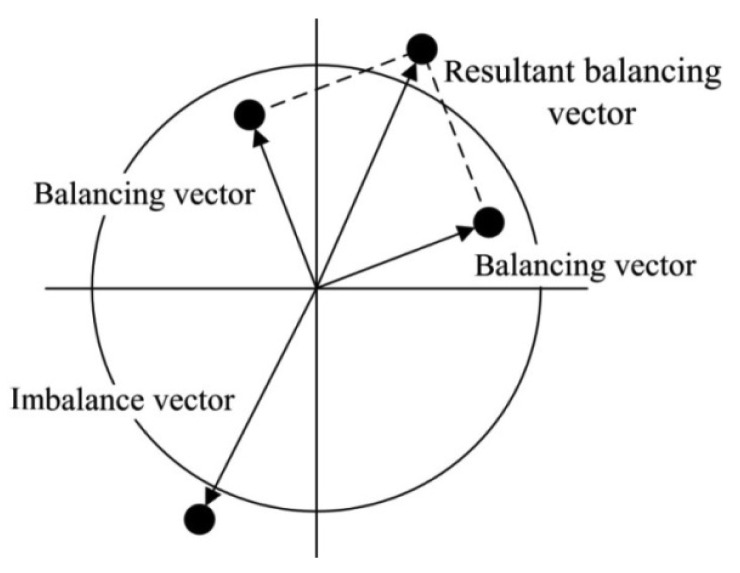
Principle of dynamic balancing.

The stator and the rotor are pre-tensioned in the radial direction to avoid an increase in the motor thickness during operations. Additionally, a counterweight block is installed on each rotor and rotates with the rotor. When the piezoelectric stator resonates in an in-plane bending vibration mode under high-frequency sinusoidal voltage excitation, the stator will act as a friction drive to move the rotor structure to the desired position to alter the balancing vector. The combined action of the two piezoelectric actuating devices creates a resultant balancing vector that achieves dynamic balance adjustment by controlling the positions of the two counterweight blocks. The dynamic balancing principle is shown in Figure 3.

**Figure 4.**
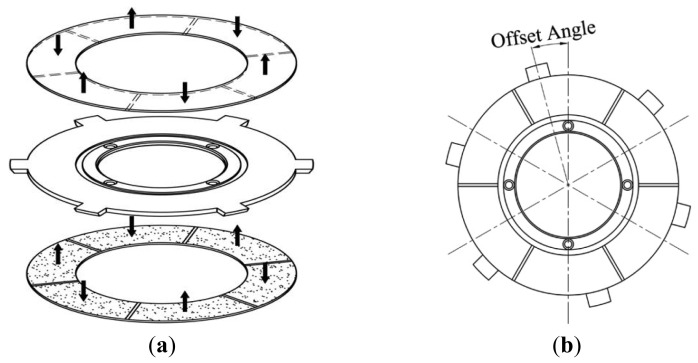
Structural diagrams of the piezoelectric stator.

The piezoelectric stator consists of an annular elastic metal body with two annular piezoceramic discs bonded to the top and bottom. Six driving teeth are distributed equally around the outer circumference of the metal body. Each bi-directionally polarised piezoceramic disc has six uniformly polarised regions, as shown in Figure 4(a). In this figure, the black arrows represent the direction of the polarisation of each region. The two identical piezoceramic discs are bonded so that the regions in the same circumferential location on the two discs have opposite polarisations. The radial centre line of each region of the piezoceramic discsis offset by a certain angle from the midline of the nearest driving tooth on the metal body, as shown in Figure 4(b).

Under the excitation of a single-phase sinusoidal voltage at a specific frequency, a single wavelength of a standing wave is induced in each pair of neighbouring regions on each piezoceramic disc; consequently, the composite piezoelectric stator is three wavelengths long in the circumferential direction. When three identical high-frequency sinusoidal electrical signals are applied to the two piezoceramic discs, respectively, using the metal body as one electrode and the unbonded surfaces of the two piezoceramic discs as the other electrode, each disc generates its own standing wave pattern according to its polarisation pattern. The two standing waves are in phase and can excite the composite piezoelectric stator to produce the desired in-plane bending vibration modes (3,1), (3,2) and (3,3), respectively. Because the (3,1) mode can be excited at relatively low frequency and has greater level radial and circumferential displacement components than the other two modes, it is chosen as the operating vibrational mode of the regulator.

**Figure 5.**
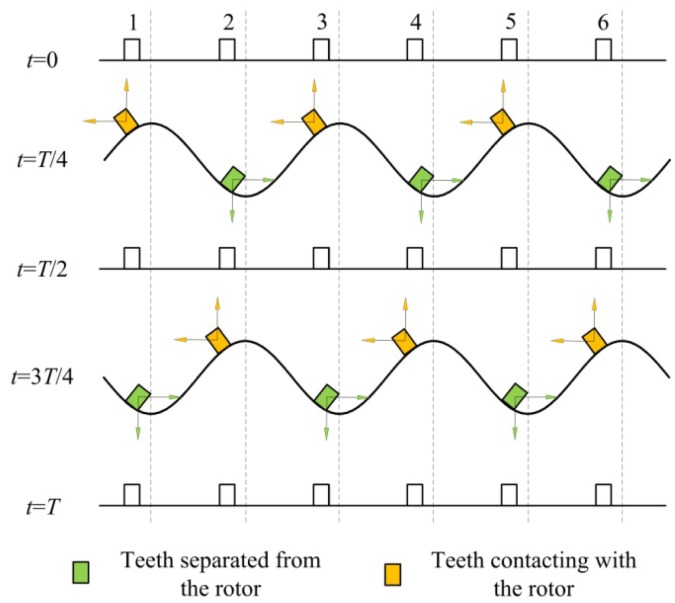
Displacement distribution diagram of the stator.

In the in-plane bending vibration mode (3,1), each point on the outer circumference of the annular stator has both radial and circumferential motion components. The circumferential displacement is non-zero for all points, except those at extreme radial displacements, *i.e.*, the crests and troughs of the stator vibration waves. Consequently, the six driving teeth are placed on the outer circumference of the annular stator so that when the teeth contact the rotor, their radial displacement produces a positive radial pressure on the rotor. Simultaneously, the circumferential displacement of the teeth produces relative motion in the rotor, and the rotor is propelled by the contact friction between the teeth and the rotor. Figure 5 shows the displacement vector of each tooth plotted with respect to its angular position, where the vibration period is *T*, the time is *t* and the outer circumference of the stator and the six driving teeth are spread along the abscissa. In the first half of the vibration period, the three teeth (1,3 and 5) in the rising state act to propel the output rotor in a counter-clockwise direction during contact. In the second half of the vibration period, the other three teeth (2, 4 and 6) act in the same driving role. Therefore, during continuous vibration, the six teeth act in two groups to alternately propel the rotor continuously in a counter-clockwise direction.

## 3. Kinematics Modelling of the Piezoelectric Dynamic Balance Regulator

Based on motion analysis in the previous section, it is obvious that the stator intermittently contacts the rotor, so the actuation direction of the regulator is unique. The analysis includes a significant number of non-linear and uncertain factors, and the whole drive process is very complicated. To simplify the model, the following assumptions are made [8,9]:

1. the rotor is a rigid body;
2. thefriction material is visco-elastic, its surface is smooth and the influence of surface roughness is neglected;
3. the Coulomb friction law is valid at the contact interface between the stator and rotor; and
4. the regulator runs in steady state (the starting and stopping processes of the regulator are not considered).

### 3.1. Motion of the Stator

**Figure 6.**
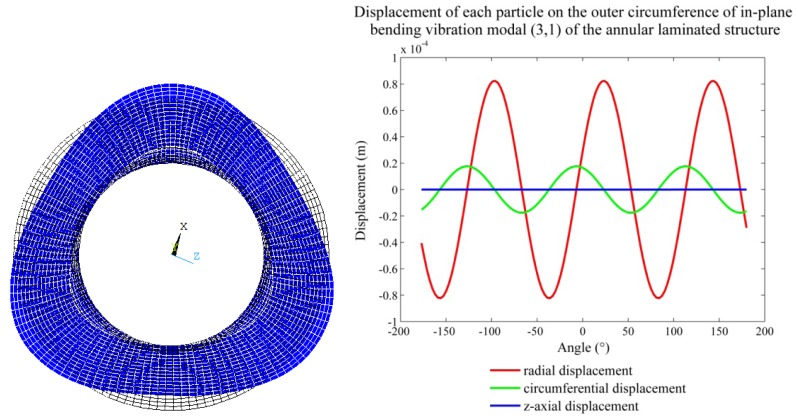
Vibration deformation diagram of the in-plane bending vibration mode (3,1) of the structure of the annular stator and the characteristics of the vibration displacement of each point on the circumference.

The displacement behaviour for the annular stator can be derived approximately using the analytical method for the in-plane vibration of the thin elastic plate [10]. First, a cylindrical coordinate system is established, with the annular stator axis as the *z*-axis and the middle annular surface of the stator as the *r*-*θ* plane. The resultant radial and circumferential displacement functions, *u_r_* and *u_θ_*, of the in-plane bending vibration mode of the annular stator structure are derived as follows [11]:

(1) $$\{\begin{array}{c} u_{r}=\left\{A\frac{\partial J_{n}\left(\text{hr}\right)}{\partial r}+B\frac{\partial Y_{n}\left(\text{hr}\right)}{\partial r}+\frac{n}{r}\left[CJ_{n}\left(\text{kr}\right)+{\text{DY}}_{n}\left(\text{kr}\right)\right]\right\}\cos(n\theta )e^{j\omega t}\\ u_{\theta }=-\left\{\frac{n}{r}\left[{\text{AJ}}_{n}\left(\text{hr}\right)+{\text{BY}}_{n}\left(\text{hr}\right)\right]+C\frac{\partial J_{n}\left(\text{kr}\right)}{\partial r}+D\frac{\partial Y_{n}\left(\text{kr}\right)}{\partial r}\right\}\sin(n\theta )e^{j\omega t} \end{array}$$

where $h={\left(\frac{\rho \omega^{2}}{\lambda +2G}\right)}^{\frac{1}{2}}$, $k={\left(\frac{\rho \omega^{2}}{G}\right)}^{\frac{1}{2}}$, $\lambda =\frac{\mu E}{\left(1-\mu^{2}\right)}$, $G=\frac{E}{2\left(1+\mu \right)}$, *λ* is Lamé's constant, *μ* is Poisson's ratio, *E* is Young's modulus, *G* is the shear modulus, *ρ* is the material density, *ω* is the resonant angular frequency, *n* is the number of the wave or nodal diameter of the in-plane vibration, *A*, *B*, *C* and *D* are coefficients determined by the boundary conditions of the annular plate structure and *J_n_* and *Y_n_* are Bessel functions of the first and second type of the *n*-th order, respectively. The annular stator is considered to be a laminated annular plate with clamped boundary conditions at the inner edge and free boundary conditions at the outer edge. The boundary conditions are adopted to derive the resonant frequency of the in-plane vibration mode of the piezoelectric stator and are as follows:

(2) $$\{\begin{array}{c} {T_{\text{rr}}|}_{r=a}=\left(\lambda +2G\right)\frac{\partial u_{r}}{\partial r}+\lambda \left(\frac{1}{r}\frac{\partial u_{\theta }}{\partial \theta }+\frac{u_{r}}{r}\right)=0;\\ {T_{r\theta }|}_{r=a}=G\left(\frac{\partial u_{\theta }}{\partial r}+\frac{1}{r}\frac{\partial u_{r}}{\partial \theta }+\frac{u_{\theta }}{r}\right)=0.\\ {u_{r}|}_{r=b}=0;\\ {u_{\theta }|}_{r=b}=0; \end{array}$$

where *a* and *b*are the outer and inner radii of the annular stator structure, respectively, and *T_rr_* and *T_rθ_* are the radial normal stress and tangential shearing stress, respectively. Substituting the displacement function in Equation (1) into the boundary conditions in Equation (2), the resonant frequency *f_r_* for the annular stator is:

(3) $$f_{r}=\frac{\alpha_{n,m}}{2\pi a}\sqrt{\frac{E}{\rho \left(1+\mu^{2}\right)}}$$

where *α_n,m_* is the natural frequency constant of the annular plate, the subscript *m* represents the number of the nodal circle and *n* represents the number of the wave or nodal diameter. The ANSYS finite element software is used to analyse the corresponding modal shapes of the in-plane bending vibration of the piezoelectric stator, as shown in Figure 6. The structural dimensions of the FEM model are shown in Table 1, and the material parameters are shown in Table 2.

In Figure 6, the whole circumference is outspread as a sine curve, and the displacement of the outer circumference of the stator can be given by:

(4) $$\{\begin{array}{c} u_{r}\left(x,t\right)=u_{\text{rmax}}\sin\left(\text{nx}\right)\sin\left(\omega t\right)\\ u_{\theta }\left(x,t\right)=u_{\theta \text{max}}\cos\left(\text{nx}\right)\sin\left(\omega t\right) \end{array}$$

where *u_rmax_* and *u_θmax_* are the maximum radial and circumferential displacements, respectively.

**Figure 7.**
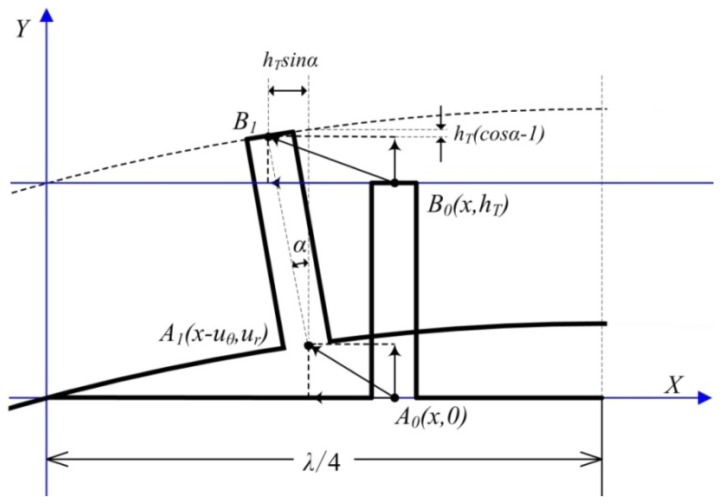
Local deformation diagram of the in-plane bending vibration mode (3,1) of the stator.

To simplify the analysis, an orthogonal coordinate system is set up, as shown in Figure 7. A local spread diagram expanded in the circumferential direction of the stator is drawn in the coordinate system, where λ is the wavelength of the standing wave.

Two points are employed to perform the dynamic analysis and are located at the root and top of the stator tooth, respectively (the two points are called the root point and the top point in this paper, respectively). The variables *A_1_* and *A_0_* are the positions of the root point with and without deformation, respectively. The variables *B_1_* and *B_0_* are the positions of the top particle with and without deformation, respectively. The variable *α* is the swing angle of the tooth, and *h_T_* is the length of the tooth. The deformed position*A_1_*(*x-u_θ_, y+u_r_*) is displaced from the initial position *A*(*x, y*) by (*u_θ_,u_r_*) when the piezoceramic stator is excited by a sinusoidal electric signal. The general expressions for the vertical deformation *w_r_* and the horizontal deformation *w_θ_* of the top pointare given as Equations (5) and (6), respectively:

(5) $$w_{r}(x,t)=u_{r}(x,t)-h_{T}(\cos\alpha -1)$$

(6) $$w_{\theta }(x,t)=u_{\theta }(x,t)+h_{T}\sin\alpha$$

For small values of *α*, the following approximations hold true:

(7) $$\{\begin{array}{c} \cos\alpha \approx 1\\ \sin\alpha \approx \tan\alpha \end{array}$$

Accordingly, Equations (5) and (6) can be simplified as follows:

(8) $$w_{r}(x,t)\approx u_{r}(x,t)$$

(9) $$w_{\theta }(x,t)\approx u_{\theta }(x,t)+h_{T}\tan\alpha$$

Additionally, the swing angle *α* can be given as:

(10) $$\tan\alpha =\frac{\partial u_{r}(x,t)}{\partial x}$$

According to the above analysis, the vertical deflection of outer circumferential surface of the stator can be expressed as:

(11) $$u_{r}(x,t)=u_{\text{rmax}}\sin(nx)\sin(\omega t)$$

Substituting Equation (11) into (10), the following expression is obtained:

(12) $$\begin{array}{c} \tan\alpha =u_{r\max}n\cos n(x-u_{\theta })\sin\omega t\\ =u_{r\max}n(\cos nx\cos nu_{\theta }+\sin nx\sin nu_{\theta })\sin\omega t \end{array}$$

For sin*nu_θ_* ≈ *nu_θ_,* and cos*nu_θ_* ≈ 1, Equation (12) becomes:

(13) $$\tan\alpha \approx u_{r\max}n(\cos nx+nu_{\theta }\sin nx)\sin\omega t$$

For this case, *nu_θ_*sin*nx* ≪ cos*nx*, and Equation (13) can be rewritten as:

(14) $$\tan\alpha \approx u_{r\max}n\cos\text{nx}\sin\omega t$$

According to Equation (9), the tangential velocity of the top point can be obtained from:

(15) $$v_{T}=\frac{\partial w_{\theta }(x,t)}{\partial t}$$

### 3.2. Motion of Particles on Surface of Friction Layer

In the piezoelectric dynamic balance regulator, the high-frequency intermittent friction contact between the stator and the rotor is essential to generate the driving force. Tribological processes occurring in high-frequency friction contacts determine the torque-speed characteristics, lifetime and long-term behaviour of the regulator. The appropriate choice of materials for the stator-rotor interface is important in the design of the regulator. Until now, intermittent-contact type ultrasonic motors have successfully used hard contact materials such as Al_2_O_3_. However, in this paper, a special hard composite material coating is proposed for the regulator, which is softer than the base material and has a high wear resistance and stable mechanical properties with respect to temperature and environmental changes. The composite material employs an epoxy resin as base material, MoS_2_ and Al_2_O_3_ as padding materials. The curing temperature is possessed at 80 °C.

If the contact mechanics are studied assuming that the vibration characteristics of the stator are not affected by the contact processes, deformation will be produced on the friction layer when the rotor is pressed against the stator. The intermittent contact condition is assumed during steady state, and thus, there is not a contact gap between the interface at the driving teeth and the friction layer. Therefore, the displacement in the y-direction at a point on the friction layer is equal to the y-direction displacement of the top point. The y-direction displacement *w_Cr_* and the linear velocity in the y-direction of the top point can be derived as follows:

(16) $$w_{\text{Cr}}(t)=w_{r}(x,t)$$

(17) $$v_{\text{Cr}}=\frac{{\mathrm{dw}}_{Cr}(t)}{\text{dt}}$$

As mentioned above, the rotor is assumed to be a rigid body. Additionally, the friction layer solidifies directly on the inner circumferential surface of the rotor. Therefore, the angular velocity of the rotor is equal to the angular velocity of the friction layer:

(18) $$\omega_{C\theta }=\omega_{R}$$

where *ω_R_* and *ω_Cθ_* are the angular velocities of the rotor and the friction layer, respectively.

### 3.3. Motion of the Rotor

Because the friction layer is softer than the base material of the rotor, the contact deformation is limited to the friction layer region. Thus, the rotor can be seen as a rigid body rotating on a fixed axis, and the equation describing the rotational inertia of the rotor can be given as:

(19) $$J_{R}=\sum {m_{i}r_{i}}^{2}$$

where *m_i_* is the quality of the point of the rotor, *r_i_* is the rotational radius of the point *m_i_* and *J_R_* is the sum of the inertia moments of the rotor.

The momentum equation for a rigid rotor rotating on a fixed axis is given as:

(20) $$J_{R}\frac{d\omega }{\text{dt}}=\sum M(\vec{F})$$

where the right side of the equation expresses the momentum sum of all the external forces.

The dynamic differential equation describing the rotor used in this research is written as:

(21) $$J_{R}\frac{d\omega_{R}}{\text{dt}}=M_{D}-M_{L}$$

where *M_D_* is the driving torque from the stator and *M_L_* is the load torque.

### 3.4. Stator-Rotor Contact Model Analysis

The mechanics of the stator-rotor contact for the regulator are complicated due to the many parameters that must be taken into account. For the case of the rotor being regarded as a rigid body and the motion of the stator is assumed independent of the contact conditions, a visco-elastic foundation model can be employed to describe the stator-rotor contact characteristics. In the proposed regulator, the normal contact resultant force *F_N_* between the stator and rotor consists of two parts; one is the pre-load force *F_C0_* between the stator and the rotor, and the other is generated by means of in-plane vibration waves from the piezoceramic stator. In this paper, we restrict our attention to the contact problem between the stator and the rotor. For simplicity, we assume that the in-plane vibration waves are generated by a suitable distributed force *F_CN_* acting on the stator [12]. The normal contact resultant force is therefore given as:

(22) $$F_{N}=F_{C0}+F_{\text{CN}}$$

where the units of *F_C0_* and *F_CN_* are N/m.

To further simplify the analysis, the essential assumption that the distributed contact force locally depends only on the local deformation at the contact points is used. The Kelvin-Voigt constitutive equation is therefore used to describe the stress-strain relationship of the friction material and is given by:

(23) $$\sigma (t)=E_{C}\varepsilon (t)+\vartheta_{C}\frac{d\varepsilon (t)}{\text{dt}}$$

where *E_C_* and ϑ_C_ are the Young's modulus and the viscosity coefficient of the friction material, respectively. The variables *σ*(*t*) and *ε*(*t*) are the stress and the strain of the friction layer, respectively. Additionally, *ε*(*t*) can be given as:

(24) $$\varepsilon (t)=\frac{u_{\text{CN}}(t)}{h_{C}}$$

where *h_C_* is the thickness of the friction layer.

For a contact area with a width of *b_C_*, the normal contact force *F_CN_*(*t*) is obtained as:

(25) $$F_{\text{CN}}(t)=b_{C}\sigma (t)$$

Substituting Equation (23) into (25), the following expression is obtained:

(26) $$F_{\text{CN}}(t)=\frac{E_{C}b_{C}}{h_{C}}u_{\text{CN}}(t)+\frac{\vartheta_{C}b_{C}}{h_{C}}\frac{d}{\text{dt}}u_{\text{CN}}(t)$$

Because the friction forces depend mainly on factors such as the coefficient of static/dynamic friction, and considering the forces and stresses at the interface between the stator and the rotor, the normal contact pressure and the relative motion of interfaces, two different types of “friction” should be distinguished [8,13].

The maximum tangential resisting force at the contact interface before sliding begins is called the maximum static friction force *f_S_* and in most cases *f_S_* is proportional to the normal force *F_N_*:

(27) $$f_{s}=\mu_{s}F_{N}$$

where *μ_S_* is the coefficient of static friction.

Once relative motion between the stator and the rotor begins, a certain tangential resistance force *f_d_*, called the dynamic friction force, arises at the contact interface. In most cases, *f_d_* is also proportional to the normal force *F_N_*:

(28) $$f_{d}=\mu_{d}F_{N}$$

where *μ_d_* is the coefficient of dynamic friction.

**Figure 8.**
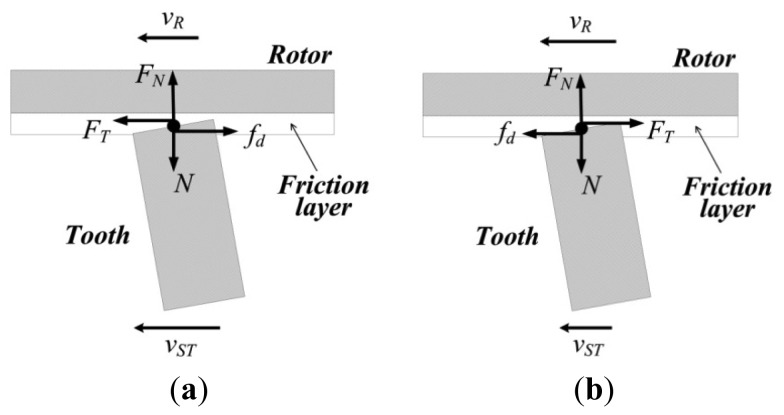
Diagram of contact between the stator and the rotor.

At the contact interface, while the linear velocity of the points on top of the driving teeth are greater than the corresponding points on the surface of the friction layer, the rotor is driven by the stator, and it can be thought that the friction force creates positive work, as shown in Figure 8(a). Conversely, the rotor is slowed by the stator and the friction force can be thought to create negative work, as shown in Figure 8(b). Thus, the tangential forces on rotor can be given as:

(29) $$F_{T}=\{\begin{array}{cc} \mu_{d}F_{N} & v_{R}<v_{\text{ST}}\\ -\mu_{d}F_{N} & v_{R}>v_{\text{ST}} \end{array}$$

where *ν_ST_* and *ν_R_* are the linear velocities of the top point in the x-direction and the corresponding point on surface of the friction layer, respectively.

Because the piezoelectric stator has six drive teeth and makes use of only three teeth to drive the rotor, according to the Coulomb friction law, the driving torque *M_D_* can be therefore calculated as:

(30) $$M_{D}=3r_{p}F_{T}$$

where *r_p_* is the inner radius of rotor.

### 3.5. Energy Conversion Characteristics of the Regulator

A common feature of all piezoelectric actuators is their two-stage energy conversion process. In the first stage, the piezoelectric elements convert electric power energy to mechanical vibration power by the piezoelectric ceramics employed in the stator to induce standing waves in the stator at frequencies in the ultrasonic range. In the second stage, the wave energy in the stator is transferred to the rotor by means of the friction contact force between them. The energy conversion efficiency of the second stage is discussed in detail in this paper. Because the intermittent contact between the stator and the rotor is cyclical, it is possible to choose a contact cycle to analyse the energy conversion characteristics [13]. The power transferred to the rotor is:

(31) $$P_{\text{out}}=\int_{T}^{t+T}M_{L}\omega_{R}\text{dt}$$

The frictional losses at the interfaces are:

(32) $$P_{\text{loss}}=\int_{T}^{t+T}\left(nL_{C}\left|F_{T}\right|\cdot \left|v_{\text{ST}}-v_{\text{CT}}\right|\right)\text{dt}$$

where *L_C_* is the length of the contact area between a stator tooth and the rotor.

The energy dissipation from damping in the friction layer is:

(33) $$P_{\text{diss}}=\int_{T}^{t+T}\left\{nL_{C}\left[C_{S}{\dot{w}}_{\theta }(t)v_{\text{ST}}+C_{S}{\dot{w}}_{r}(t)v_{\text{SN}}\right]\right\}\text{dt}$$

where *C_S_* is the equivalent damping coefficient of the friction material.

Finally, the conversion efficiency of the interface can be given by:

(34) $$\eta =\frac{P_{\text{out}}}{P_{\text{out}}+P_{\text{loss}}+P_{\text{diss}}}$$

## 4. Numerical Simulation and Test

### 4.1. Effects of Some Parameters on Regulator Characteristics

In the proposed research on the piezoelectric dynamic balance regulator, the qualitative relationships between the structural parameters and the energy conversion characteristics must be determined. Therefore, MATLAB is employed to numerically simulate characteristics of the regulator based on the above mathematical model. The model parameters are found in Table 3.

When some parameters are analysed, they do not use the values from Table 1, but vary between certain limits. In the following analysis, *P_out_* is the output power and (*P_loss_* + *P*diss) as a whole is the loss power.

#### 4.1.1. Effects of Dynamic Friction Coefficient on Regulator Characteristics

**Figure 9.**
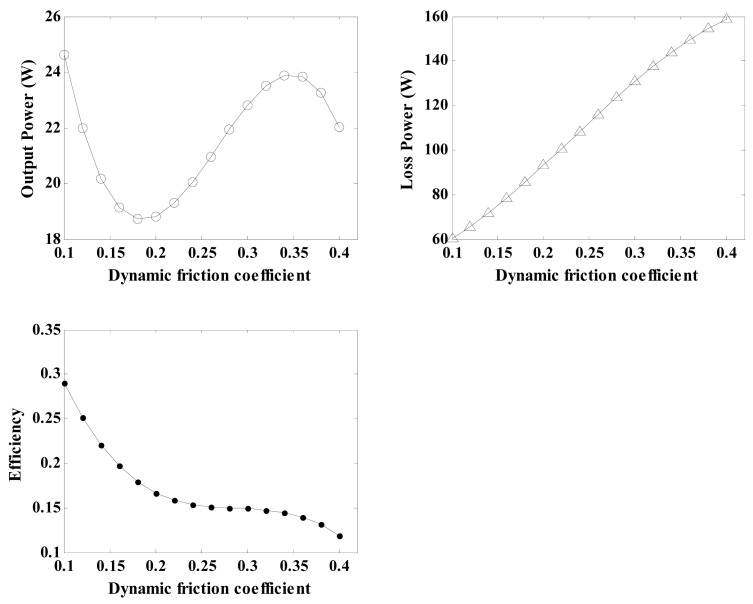
Diagram of the effects of the coefficient of dynamic friction on the regulator characteristics.

The coefficient of dynamic friction is one of the most important properties of the friction material, and it is necessary to analyse its influence on the output characteristics of the regulator. By using the related parameters of the regulator depicted in Table 3 and Equations (21), (29), (30), (31), (32), (33) and (34), the relationship between the coefficient of dynamic friction and the regulator characteristics can be calculated and shown in Figure 9. With an increase in the coefficient of dynamic friction, the loss power exhibits nearly linear growth, and the conversion efficiency curve remains approximately unchanged for coefficients of dynamic friction varying from 0.25 to 0.35. Therefore, a friction material possessing a coefficient off riction in the above range is the best choice for this analysis.

#### 4.1.2. Effects of Young's Modulus of Friction Material on Regulator Characteristics

Young's modulus is also an important property of the friction material, and it is important to research its effects on the regulator characteristics. By using the related parameters of the regulator depicted in Table 3 and Equations (21), (26), (27), (30), (31), (32), (33) and (34), the relationship between the Young's modulus and the regulator characteristics can be calculated.

**Figure 10.**
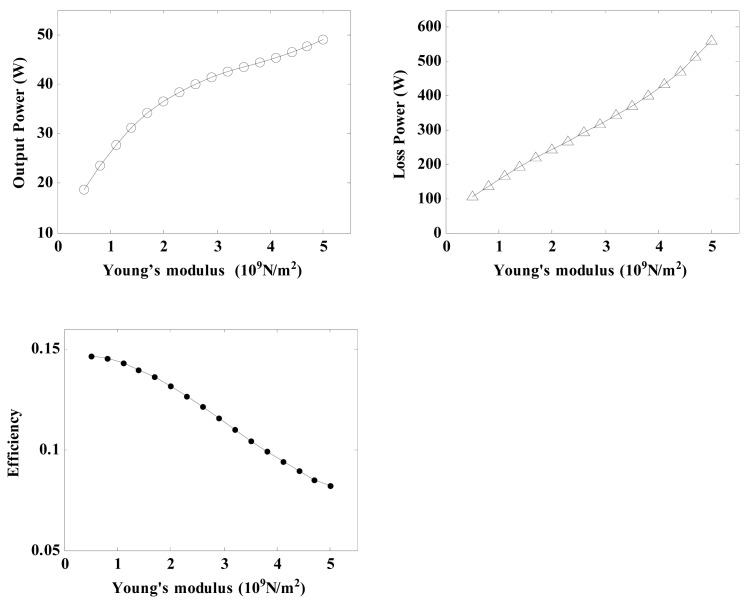
Diagram of the effects of the Young's modulus of the friction material on the regulator characteristics.

As a result, plots of the output power, loss power and conversion efficiency *versus* the Young's modulus are shown in Figure 10. It can be seen that an increase in the elastic modulus causes the power output and the loss power to increase at an accelerated rate, whereas the conversional efficiency falls gradually. As a result, if a relatively value for high conversion efficiency is demanded, the Young's modulus of the friction material should be chosen to be between 1 × 10^9^ and 2 × 10^9^ N/m^2^.

#### 4.1.3. Effects of Thickness of Friction Layer on the Regulator Characteristics

**Figure 11.**
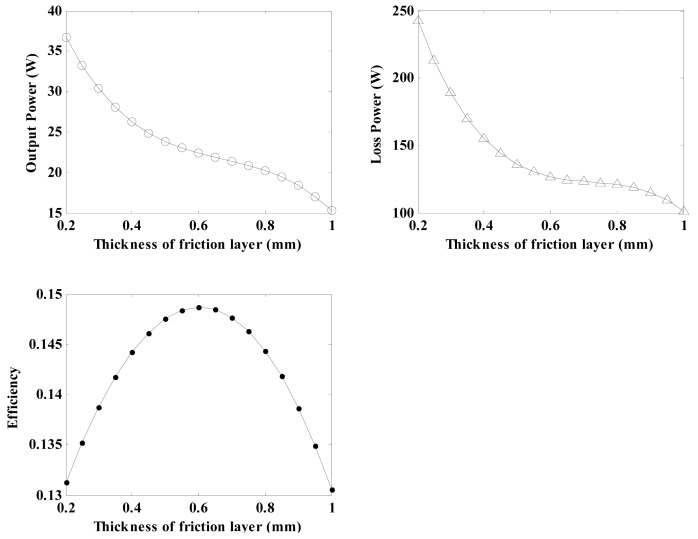
Diagram of the effects of the thickness of the friction layer on the regulator characteristics.

By using the related parameters of the regulator depicted in Table 3 and Equations (21), (26), (29), (30), (31), (32), (33) and (34), the relationship between the Young's modulus and the regulator characteristics can be calculated. As a result, the output power, loss power and conversion efficiency for the regulator plotted *versus* the thickness of the friction layer (varying between 0.2 mm and 1 mm) are shown in Figure 11. It can be seen that as the thickness increases, the output power and loss power show similar downward tendencies. The conversion efficiency exhibits significant higher values when the thickness of the friction layer varies between 0.5 mm to 0.7 mm. On the basis of these analyses, it is confirmed that the friction layer thickness should be controlled to obtain higher conversion efficiencies. Therefore, 0.6 mm is chosen to be the thickness of the friction layer in this research.

#### 4.1.4. Effects of Pre-Load Force on the Regulator Characteristics

**Figure 12.**
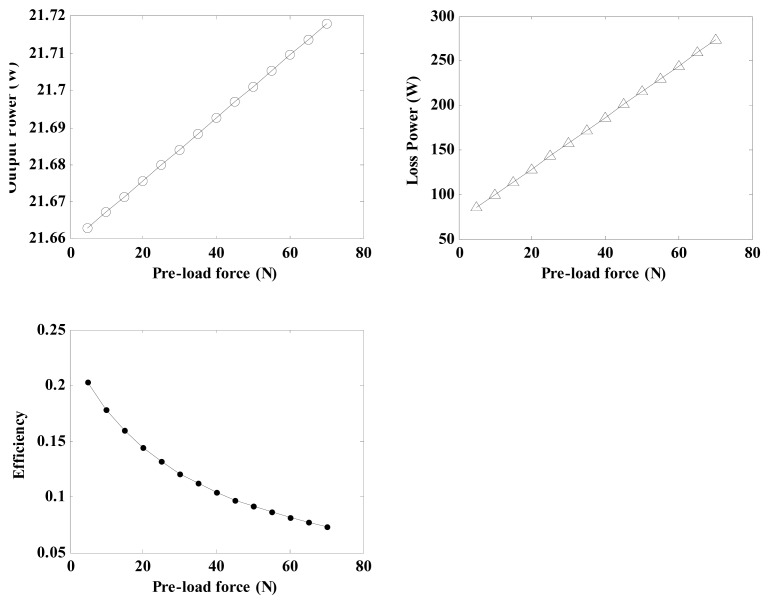
Diagram of the effects of the pre-load force on the regulator characteristics.

By using the related parameters of the regulator depicted in Table 3 and Equations (21), (22), (29), (30), (31), (32), (33) and (34), the relationship between the pre-load force and the regulator characteristicsfor a friction layer thickness of 0.6 mm can be calculated and shown in Figure 12. The plots indicate that the output power and loss power increase with the pre-load force in a certain range. Simultaneously, the conversion efficiency decreases gradually. High pre-load forces not only induce wear but also produce stall torques that result in the regulator becoming jammed. A value of 20 N is chosen for pre-load force in this research.

#### 4.1.5. Effects of Load Torque on Regulator Characteristics

**Figure 13.**
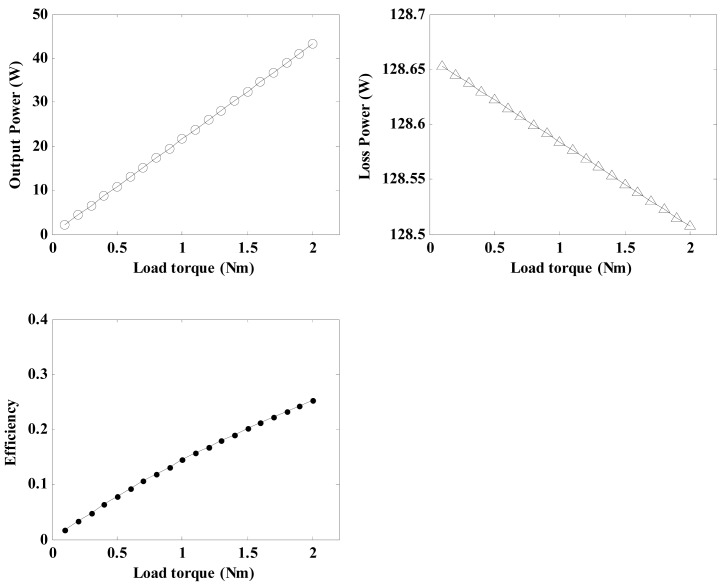
Diagram of the effects of the load torque on the regulator characteristics.

By using the related parameters of the regulator depicted in Table 3 and Equations (21), (31), (32), (33) and (34), the relationship between the load torque and the regulator characteristics for a pre-load force of 20 N and a friction layer thickness of 0.6 mm can be calculated and shown in Figure 13.

It can be seen that the output power and conversion efficiency show similar upward trends with an increase in the load torque. However, the loss power decreases gradually. Therefore, an increase in the load torque will not increase the loss power, which is produced by the friction between the stator and the rotor.

### 4.2. Comparison between Simulated and Test Results

**Figure 14.**
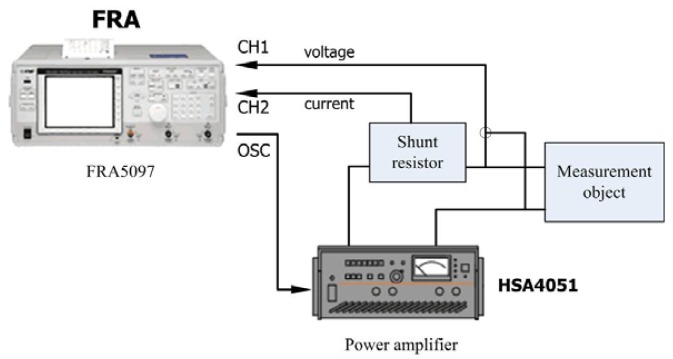
Schematic of the experimental measurement of the resonant frequency response of the piezoelectric stator.

**Figure 15.**
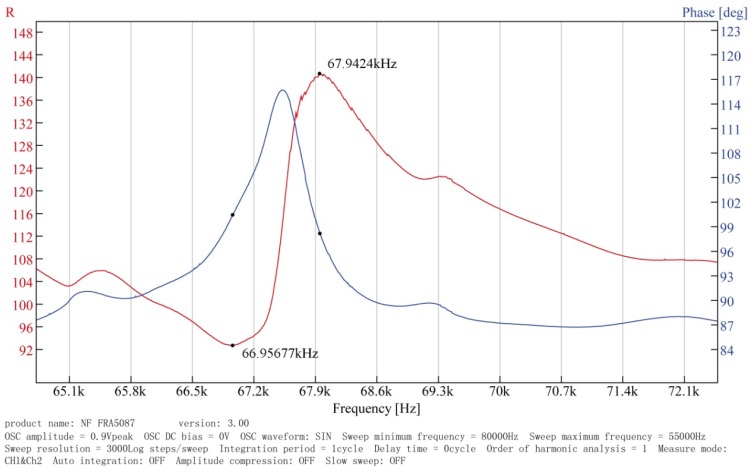
Impedance characteristics of the regulator in the 55 kHz to 80 kHz frequency band.

Through the analysis of the dynamic model of the regulator by means of MATLAB, the simulation results allow one to determine the key parameters of the regulator. Using the parameters shown in Table 2, a prototype was fabricated, and its performance was tested experimentally. To verify the analytical and numerical modal analyses of the regulator, an experimental test bench is established primarily to measure the operating frequency of the in-plane bending vibration of the piezoelectric stator, as shown in Figure 14. The test bench includes a frequency response analyzer FRA5097 to generate the excitation signal, a power amplifier HSA4051 to amplify the signal to excite the stator, and both instruments are products of the NF Company. The output voltage and current of the stator are measured simultaneously by the analyzer to obtain the frequency response of the impedance. The impedance-frequency characteristic of the piezoelectric stator measured in the experiment is shown in Figure 15.

**Figure 16.**
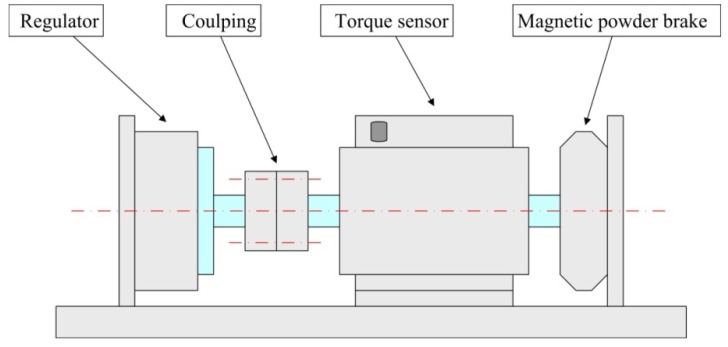
Schematic of the regulator load torque/rotational speed relational measurement test bench.

**Figure 17.**
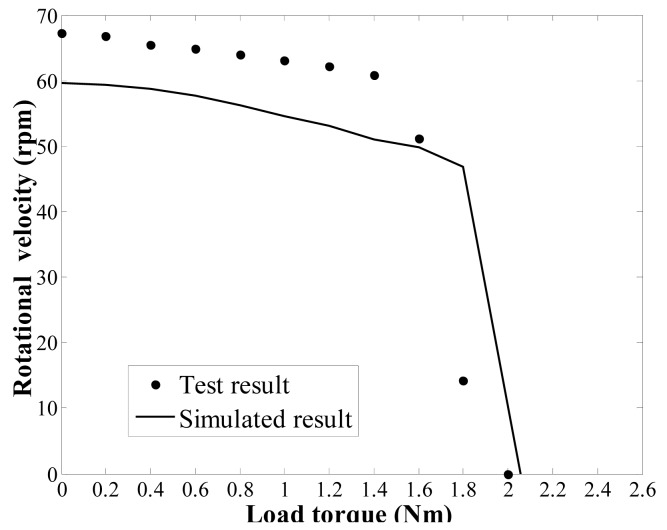
Diagram of the experimental and simulated load torque/rotational speed relation.

Figure 15 shows the impedance characteristics of the regulator. It is can be seen that the actual resonant frequency of the in-plane bending vibration mode (3,1) of the stator, *i.e.*, the operating frequency of the regulator, is approximately 67 kHz. This value is slightly higher than the computational results. After that, an experimental test bench is established to measure the load torque/rotational speed relation of the regulator, as shown in Figure 16. The test bench includes a magnetic powder brake ZX-0.3YN-24 to generate the the load torque, a torque sensor (with built-in speed sensor) JN338-A to measure the load torque and rotational speed in real time, and the two equipments are produced by the MITSUBISHI Company and BEIJING SANJING Company respectively. Figure 17 shows the experimental and simulated load torque/rotational speed relation for the regulator. The round dots are the test results, and the solid line is the simulated results which is calculated by using the related parameters of the regulator depicted in Table 3.

It can be seen that the simulation accuracy is approximately 10% lower than the measured experimental rotational speed of the regulator. The simulated result of the stall torque is approximately 3% lower than the measured experimental stall torque. Several reasons can be given for this error between experimental and simulated values. The proposed dynamic model is an approximation of the actual regulator and a parametric model in which design and optimisation processes are possible. To achieve a balance between computational efficiency and accuracy, some of the necessary assumptions may cause modelling error. For example, a possible reason for the modelling error could arise from the fact that the rotor model does not include the rotor flexion because it was assumed to behave as a rigid body. Moreover, the in-plane bending vibration waves are assumed to be generated by a distributed force acting on the stator. This approximation also causes some degree of modelling error. However, the results from the simulations and experiments generally exhibit the same trend, and thus, the validity of the dynamic model is proven. To summarise, the mathematical model presented and analysed in this paper can be used as a tool for optimising the design of a piezoelectric dynamic balance regulator in steady state operation.

## 5. Conclusions

In this paper, an ultrasonic piezoelectric dynamic balance regulator using the basic principles of a standing wave-type ultrasonic motor is proposed. Its overall structure and the actuation principle are introduced. The FEM and analytical analysis based modelling studies of the regulator are investigated in detail. The proposed model is simple and suitable for the study on friction behavior at the contact interface of regulator, and can simulate and analyze the effects of thickness and Young's modulus of friction layer, dynamic friction coefficient of contact interface, pre-load and load torque, *etc.* on the performances of regulator, and can be used to optimize design of the regulator.Based on the above model, MATLAB software is employed to numerically simulate characteristics of the regulator. Through the analysis of the dynamic model of the regulator by means of MATLAB, the simulation results allow one to determine the key parameters of the regulator. Using these parameters, a self-developed regulator prototype is fabricated, and its performance is tested experimentally. The experimental results are in good agreement with the simulation results, which demonstrates the effectiveness of the proposed method. Thus it can be seen that the numerical modelling approach to this complex problem has provided valuable insight into the mechanics and dynamics of the regulator. The model presented here lays the foundation for a general framework capable of the existing ultrasonic piezoelectric dynamic balance regulator and serving as a useful design tool for optimizing the key parameters of future regulators. The research on the model of the new piezoelectric dynamic balance regulator is still in its initial stages. For future applications of the regulator, systematic research and studies will be necessary. For example, the effects of stator teeth and the starting and stopping of the motor are not considered. These effects will be further researched in the future.

## Figures and Tables

**Table 1.** Structural dimensions of the FEM model.

**Parameter Name**	**Parameter Values**	**Unit**
Outer diameter of stator	80	mm
Inner diameter of stator	51	mm
Thickness of stator metal body	2.4	mm
Thickness of piezoelectric ceramic piece	0.5	mm

**Table 2.** Material parameters of the stator.

Material of stator mental body	Aluminium alloy	Physical properties	Young's modulus	7 × 10^10^ N/m^2^
			Material density	2,730 kg/m^3^
			Poisson's ratio	0.3
Material of piezoceramicdisk	P-81	Physical properties	Young's modulus	13.2 × 10^10^ N/m^2^
			Material density	7,450 kg/m^3^
			Poisson's ratio	0.32
			Mechanical quality factor *Q_m_*	800
		Electrical properties	Relative dielectric constant ${ɛ}_{r3}^{T}$	1,000
			Relative dielectric constant ${ɛ}_{r1}^{T}$	1,400
			Dielectric loss *tgδ*	0.5%
			Planar electromechanical coupling factor *k_p_*	0.50
			Piezoelectric strain constants of the piezoceramic *d_31_*	90 × 10^−12^ C/N
			Piezoelectric strain constants of the piezoceramic *d_33_*	200 × 10^−12^ C/N
			Piezoelectric strain constants of the piezoceramic *d_15_*	410 × 10^−12^ C/N

**Table 3.** Structural and material parameters of the regulator.

**Parameter Name**	**Unit**	**Value**
Outer diameter of stator	mm	80
Inner diameter of stator	mm	51
Outer diameter of rotor	mm	97.6
Inner diameter of rotor	mm	87.6
Width of stator tooth	mm	8
Length of stator tooth	mm	4.5
Density of stator	kg/m^3^	2,730
Number of nodal diameters, *n*		3
Resonant frequency of stator, *f_r_*	kHz	65
Vibration amplitude, *u_rmax_*	mm	0.08
Vibration amplitude, *u_θmax_*	mm	0.02
Young's modulus of metal body	N/m^2^	7 × 10^10^
Young's modulus of friction material	N/m^2^	1.5 × 10^9^
Dynamic friction coefficient of friction material		0.3
Thickness of the friction layer	mm	0.6
Viscosity coefficient of friction material	Ns/m^2^	1.86 × 10^−6^
Equivalent damping coefficient of friction material	Ns/m^2^	1,000
Pre-load force	N	20
